# Hirtinone, a Novel Cycloartane-Type Triterpene and Other Compounds from *Trichilia hirta* L. (Meliaceae)

**DOI:** 10.3390/molecules18032589

**Published:** 2013-02-26

**Authors:** Ivo José Curcino Vieira, Otoniel de Aquino Azevedo, Jucimar Jorgeane de Souza, Raimundo Braz-Filho, Milena dos Santos Gonçalves, Marcelo Francisco de Araújo

**Affiliations:** 1 Laboratório de Ciências Químicas, Universidade Estadual do Norte Fluminense Darcy Ribeiro, 28013-602, Campos dos Goytacazes, RJ, Brazil; 2 Centro Universitário São Camilo, Campus I, Rua São Camilo de Léllis 01, 29304-910 Cachoeiro de Itapemirim, ES, Brazil

**Keywords:** Meliaceae, *Trichilia hirta*, terpenoids, NMR data

## Abstract

One novel triterpene cycloartane-type, named hirtinone (**1**), six protolimonoids – nilocitin (**2**), dihydronilocitin B (**3**), melianone epimers (**4**) and (**5**), piscidinol A (**6**) and melianone lactone (**7**), one tertranortriterpenoid, hirtin (**8**), and one sesquiterpene, spathulenol (**9**), were identified in the fruits of *Trichilia hirta*. The structures were established by 1D and 2D NMR (^1^H and ^13^C-NMR, DEPTQ, ^1^H-^1^H-COSY, ^1^H-^1^H-NOESY, HSQC and HMBC), high resolution mass spectroscopy (HR-ESI-MS) and infrared (IR) spectral data.

## 1. Introduction

The Meliaceae family has attracted such a great interest among phytochemists interested in bioproduction because of its very complex and diverse chemical structures and its biological activity, mainly against insects [1,2,3,4]. The *Trichilia* genus (Meliaceae) consists of about 230 species distributed throughout tropical America, which are recognized for their significant economic importance and high commercial value. Phytochemical studies have revealed that this genus is rich in terpenoids, including triterpenes, limonoids, steroids and other terpene derivatives [3,4,5,6]. Species of this genus have been also studied for their insecticidal activities and their isolated compounds revealed complex and interesting structures, including various limonoids [5,7,8]. The isolation and structural elucidation of the two novel limonoids from the fruits of *T. hirta* collected in Espírito Santo State, Brazil, was reported by Cortez *et al.* in 1992 [9]. 

**Figure 1:**
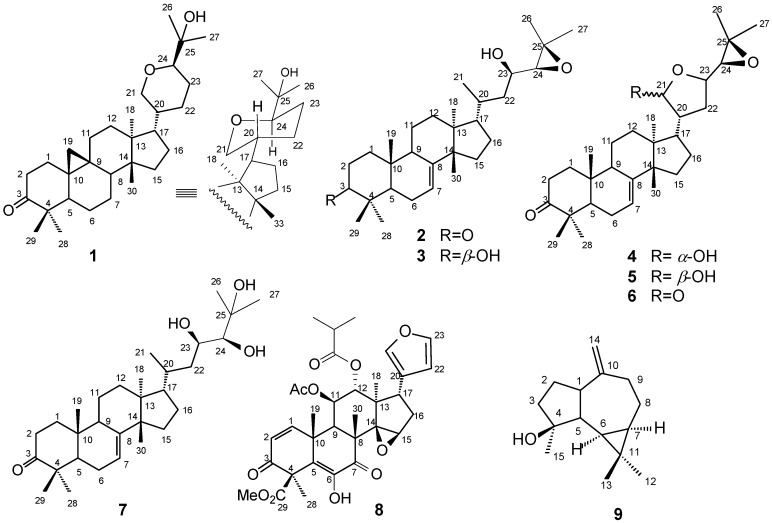
Chemical structure of the compounds isolated from the fruits of *T. hirta*.

In the present paper, we report an investigation of a hexane extract of fruits of a *T. hirta* specimen, which allowed us to characterize nine terpenoids, including a novel cycloartane-type triterpene named hirtinone (**1**), five protolimonoids: nilocitin (**2**) [10,11], dihydronilocitin B [10,11] (**3**), melianone epimers (**4**) and (**5**) [11,12], piscidinol A (**6**) [10,11,13] and melianone lactone (**7**) [11,12], the tertranortriterpenoid hirtin (**8**) [14] and the sequiterpene spathulenol (**9**) [15]. The structures were established by spectrometric techniques, mainly HRESIMS and 1D and 2D NMR, and comparative analysis with literature values. The structures of all the isolated compounds are shown in Figure 1.

## 2. Results and Discussion

The crude hexane extract of *T. hirta* fruits was submitted to chromatography and furnished nine terpenoids **1**–**9**. The known terpenoids, *i.e*., the six protolimonoids nilocitin (**2**), dihydronilocitin B (**3**), melianone epimers (**4**) and (**5**), piscidinol A (**6**) and melianone lactone (**7**), the tertranortriterpenoid, hirtin (**8**) and the sequiterpene spathulenol (**9**) were identified on the basis of ^1^H-and ^13^C-NMR spectral data, including ^1^H-^1^H-COSY, ^1^H-^1^H-NOESY, HSQC and HMBC NMR experiments, which were also used to complete and unambiguous ^1^H and ^13^C chemical shift assignments [16,17].

Hirtinone (**1**), m.p. 160–162 °C, was isolated in an amorphous form. The IR spectrum showed bands at ν_max_ 1,713, characteristic of C=O stretching of a ketone carbonyl group, as well as absorptions at ν_max_ 3,354, 2,925-2,852 and 1,089 cm^−1^, characteristics of OH , C-H and C-O stretching, respectively.

**Scheme 1:**
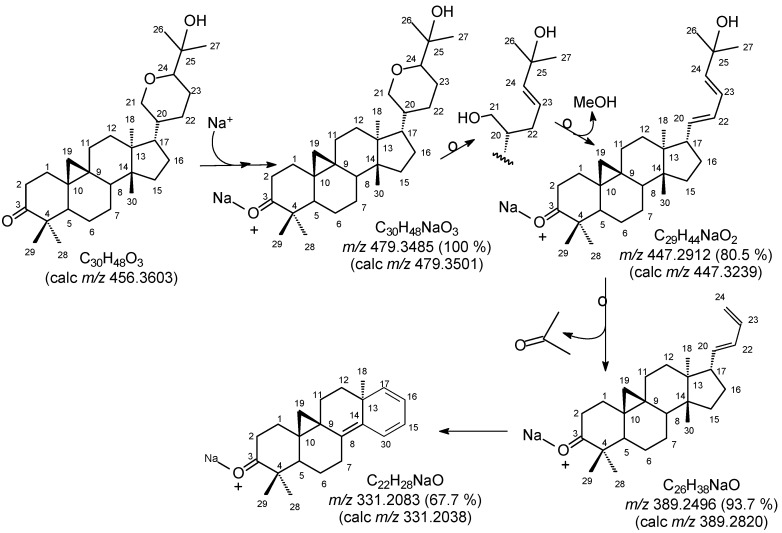
Proposed fragmentation mechanisms of **1** (only peaks classified as principal ones, with intensity of the peaks (%) in parentheses).

The HR-ESI-MS spectrum of **1** utilizing the ESI^+^ ionization mode showed a cationic base peak at *m/z* 479.3485 [M+Na]^+^, compatible with the molecular formula C_30_H_48_O_3_Na (calc. *m/z* 479.3501, Δ*_m/z_* = 0.0016). These data and the comparative analysis of the {^1^H}- and DEPTQ-^13^C NMR spectra, that allowed us to identify signals (Table 1) corresponding to seven non-hydrogenated [including one sp^2^ of carbonyl group at *δ*_C_ 216.6 (C-3) and one sp^3^ oxygenated at *δ*_C_ 71.6 (C-25)], five methine [all sp^3^ including one oxygenated at *δ*_C_ 84.1 (CH-24)], twelve methylene [all sp^3^ including one oxygenated at *δ*_C_ 72.8 (CH_2_-21)] and six methyl carbon atoms, were used to propose the molecular formula C_30_H_48_O_3_ = (C=O)(C-O)(C)_5_(O-CH)(CH)_4_(O-CH_2_)(CH_2_)_11_(CH_3_)_6_, seven unsaturation degrees (one carbonyl group in a triterpenoid with a cycloartane skeleton). The fragments postulated (Scheme 1) to justify the principal peaks observed in the HR-ESI-MS are compatible with a carbon skeleton of the cycloartane-triterpene type, including the presence of the cationized adduct as a complex involving two molecules of **1**.

The ^1^H-NMR spectra (1D and ^1^H-^1^H-COSY, Table 1) of **1**, exhibiting a cycloartane triterpene profile, showed two doublets at *δ*_H_ 0.82 (*J* = 4.1 Hz) and *δ*_H_ 0.62 (*J* = 4.1 Hz), which were characteristic for a C-9/C-10 cyclopropyl methylene and singlet signals at *δ*_H_ 0.98, 1.19, 1.16, 1.12, 1.08, 1.07 corresponding to six methyl groups. The presence of the signals at *δ*_H_ (2.72 dt, 6.4 and *J* = 13.9 Hz) and 2.34 (ddd, 1.7, 6.4 and *J* = 13.9 Hz), correlated with the ^13^C signal of a methylene carbon at *δ*_C_ 37.5 (CH_2_-2) in the HSQC spectrum indicated the presence of carbonyl group at carbon C-3, characteristic of a cycloartan-3-one triterpenoid [18,19], which was confirmed by the HMBC spectrum (Table 1) with interactions between C-3 and the 3H-28, 3H-29 and H-5 (^3^*J*_CH_) as well as H-2 (^2^*J*_H__→C_).

**Table 1:** ^1^H- (500 MHz) and ^13^C- (125 MHz) NMR of hirtinone (**1**), including results obtained by heteronuclear 2D shift-correlated HSQC and HMBC, in CDCl_3_ as solvent and TMS used as internal reference. Chemical shifts (*δ*, ppm) and coupling constants (*J*, Hz, in parenthesis) *.

		HSQC		HMBC	
Atom	Type	*δ*_C_	*δ*_H_	^2^*J*_H→C_	^3^*J*_H→C_
1	CH_2_	33.4	1.87 (m), 1.58 (m)		2H-19
2	CH_2_	37.5	2.72 (dt, 6.4; 13.9)		
			2.34 (ddd; 1.7, 6.4, 13.9)		
3	C	216.6	-	H-2a	3H-28; 3H-29; H-5
4	C	50.7	-	3H-28; 3H-29	
5	CH	48.6	1.73 (m)		2H-19; 3H-28; 3H-29
6	CH_2_	26.4	2.06 (m), 1.15 (m)		
7	CH_2_	21.5	1.60 (m), 1.05 (m)		
8	CH	49.4	1.54 (m)		3H-19; 3H-30
9	C	21.5	-	2H-19	
10	C	26.1	-	2H-19	
11	CH_2_	26.1	1.80 (m), 1.35 (m)		2H-19
12	CH_2_	35.5	1.45 (m), 1.30 (m)		3H-18
13	C	45.2	-	3H-18	3H-30
14	C	48.6	-	3H-30	3H-18
15	CH_2_	32.2	1.60 (m)		3H-30
16	CH_2_	26.6	1.40 (m), 1.15 (m)		
17	CH	47.9	1.62 (m)		3H-18
18	CH_3_	18.5	0.98 (s)		
19	CH_2_	29.6	0.82 (dl, 4.1), 0.62 (d, 4.1)		
20	CH	39.2	1.55 (m)		
21	CH_2_	72.8	4.21 (dl, 11.6), 3.06 (t, 11.6)		
22	CH_2_	30.1	1.95 (m), 1.10 (m)		
23	CH_2_	25.9	1.55 (m), 1.38 (m)		
24	CH	84.1	3.03 (dd, 11.4, 2.0)		3H-26; 3H-27
25	C	71.6	-	3H-26; 3H-27	
26	CH_3_	26.1	1.19 (s)		3H-22
27	CH_3_	24.0	1.16 (s)		3H-26
28	CH_3_	20.8	1.12 (s)		3H-24
29	CH_3_	19.2	1.07 (s)		3H-28
30	CH_3_	22.2	1.08 (s)		

* Number of hydrogens bound to carbon atoms deduced by comparative analysis of HBBD- and DEPTQ-^13^C NMR spectra. Chemical shifts and coupling constants (*J*) obtained from 1D ^1^H-NMR spectrum. Superimposed ^1^H signals are described without multiplicity and chemical shifts deduced by HMQC, HMBC and ^1^H-^1^H-COSY spectra.

The signals at *δ*_H_ 4.21 (dl, 11.6 Hz, H-21eq), 3.06 (t, 11.6 Hz, H-21ax) and 3.03 (dd, 11.4 and 2.0 Hz, H-24) were attributed to hydrogen atoms of oxymethylene (CH_2_-21) and oxymethine (CH-24), respectively, characteristic of the tetrahydropyran ring present in protolimonoid triterpenes isolated from *Trichilia* species [8,20]. The absorptions at 1713 cm^−1^ (carbonyl group), 1089 cm^−1^ (carbon-oxygen bond) and 3354 cm^−1^ (OH) observed in the IR spectrum corroborate the NMR data. The coupling constant values *J* = 11.6 Hz and *J* = 11.4 Hz observed in the signals of hydrogens H-21ax (triplet at *δ*_H_ 3.06) and H-24 (dd at *δ*_H_ 3.03) indicated an axial-axial interaction and, consequently, were used to define the axial positions of these H-20 and H-24 [20]. The analysis of the HSQC (^1^*J*_H→C_) experiment support the ^1^H- and ^13^C-NMR data and led to assignment of six methyl signals at *δ*_C_/*δ*_H_ 18.5/1.07(s), 26.1/1.19(s), 24.0/1.16(s), 20.8/1.12(s), 19.2/0.98(s) and 22.2/1.08(s)] as well as signals of methylene OCH_2_-21 [*δ*_C_/*δ*_H_ 72.8/4.21 and 3.06), methine OCH-24 (*δ*_C_/*δ*_H_ 84.1/3.03) and methylene CH_2_-19 (*δ*_C_/*δ*_H_ 29.6/0.82 and 0.62), which are compatible with the presence of tetrahydropyran ring [20] and cyclopropane ring in the compound **1** [18,19]. The presence of the cyclopropane ring was confirmed by HMBC spectrum analyses which showed cross-peaks 2H-19/C-9, 2H-19/C-10. The cross-peak at ^2^*J*_H→C_ 3H-26/C-25 and 3H-27/C-25 supporting the proposal of hydroxyl group observed in the IR spectrum (Table 1).

The relative stereochemistry of **1** was determined from the coupling constants of relevant hydrogens, from the observed ^1^H-^1^H-NOESY and from the comparison with data of analogous compounds with configuration described in the literature [20]. The relative stereochemistry of carbon atoms CH-20 and CH-24 (*vide supra*) was defined by coupling constants *J* = 11.6 Hz and *J* = 11.4 Hz observed in the signals of H-21ax (a triplet by coupling *geminal* with H-21eq–^2^*J*_HH_ and *vicinal* with H-20–^3^*J*_HH_) by and H-24 ^2^*J*_HH_ with H-23ax) and comparison with the literature ^13^C-NMR values of bourjotinolone A, a triterpene isolated from *T. hispida* [20], with both *a*-orientations being consistent with the relative configuration shown in **1.** Consistent with these observations, the ^1^H-^1^H-NOESY spectrum of **1** showed cross-peaks assigned to dipolar interaction (spatial proximity). Thus, the structure of the new triterpene cycloartane-type, isolated from *Trichilia hirta* was established as 19-methylene-25-hydroxy-20,24-oxidecycloartan-3-one named hirtinone (**1**).

## 3. Experimental

### 3.1. General Procedures

Melting points were obtained on a Microquímica MQRPF and were uncorrected. FTIR spectra were recorded on a FTIR-8300 Shimadzu spectrometer using KBr disk. ESI-MS (high resolution) mass spectra were obtained by using a micrOTOF-QII (Bruker) mass spectrometer, using the positive ion mode of analysis. Chromatographic purifications were carried out by using silica gel 60 (0.063–0.200 mm).

^1^H and ^13^C-NMR spectra were measured on a Bruker Utrashield 500 Plus spectrometer, operating at 500 (^1^H) and 125 (^13^C) MHz. CDCl_3_ was used as solvent with TMS as internal reference. Chemical shifts are given in the *δ* scale (ppm) and coupling constants (*J*) in Hz. One dimensional (1D) ^1^H and ^13^C-NMR spectra were acquired under standard conditions by using a direct detection 5 mm ^1^H/^13^C dual probe. Standard pulse sequences were used for two dimensional spectra by using a multinuclear inverse detection 5 mm probe with field gradient.

### 3.2. Plant Material

Fruits of *Trichilia hirta* were collected in May 2011, at Vale do Rio Doce Company, Linhares City, Espírito Santo State, Brazil. After botanical identification by botanist Domingos Folly. The voucher specimen of *T. hirta* was deposited at Vale do Rio Doce herbarium, under the code CRVD-6784. 

### 3.3. Extraction and Isolation

Fruits of *T. hirta* were dried at room temperature until a constant weight was achieved (about one week). The dried and powdered fruits (438.0 g) were extracted with hexane (volume, time) at room temperature, furnishing, after solvent evaporation, 23.0 g of crude hexane extract. The hexane extract was chromatographed over a silica gel column with a gradient of hexane/ethyl acetate to afford nine fractions. Fraction 8 (1.36 g) was rechromatographed over a silica gel column with a gradient of hexane/acetone furnishing eighth fractions and **6** (52.5 mg). Fractions 8.2 (23.8 mg) and 8.3 (524.2 mg) were rechromatographed over a silica gel column with a gradient of hexane/acetone yielding compounds **1** (3.1 mg) and **9** (22.5 mg), respectively. Fraction 8.5 (49.5 mg) was rechromatographed over a silica gel column with a gradient of hexane/acetone (8:2) *v/v* furnishing compound **2** (4.5 mg). Fraction 8.7 (99.3 mg) was rechromatographed over a silica gel column with a gradient of hexane/acetone furnishing compound **3** (18.5 mg). Fraction 9 (4.03 g) was rechromatographed over a silica gel column with a gradient of hexane/acetone to provide compounds **7** (67.0 mg), **4** + **5** (176.0 mg) and **8** (65.7 mg).

*Nilocitin* (**2**): ^13^C *δ* (ppm): 38.56 (CH_2_-1); 34.93 (CH_2_-2); 216.96 (C-3); 47.84 (C-4); 52.34 (CH-5); 24.36 (CH_2_-6); 118.04 (CH-7); 145.72 (C-8); 48.47 (CH-9); 36.05 (C-10); 18.86 (CH_2_-11); 33.52 (CH_2_-12); 43.35 (C-13); 51.23 (C-14); 34.06 (CH_2_-15); 29.77 (CH_2_-16); 53.28 (CH-17); 21.78 (CH_3_-18); 12.80 (CH_3_-19); 33.69 (CH-20); 19.89 (CH_3_-21); 40.47 (CH_2_-22); 69.25 (CH-23); 68.46 (CH-24); 60.32 (C-25); 24.88 (CH_3_-26); 19.82 (CH_3_-27); 24.53 (CH_3_-28); 21.61 (CH_3_-29); 27.40 (CH_3_-30). ^1^H *δ* (ppm): (2.02; 1.50; 2H-1); (2.78 *dt* 14.6, 5.6; 1H-2); (2.26 *dt* 14.6, 3.8; 1H-2) (1.70; 1H-5); (2.10; 2H-6); (5.33 *m*; 1H-7); (2.31; 1H-9); (1.60; 2H-11); (1.65; 1.40; 2H-12); (1.85; 1.55; 2H- 15); (2.08; 1.18; 2H-16); ( 1.50; 1H-17); (0.83 *s*; 3H-18); (1.03 *s*; 3H-19); (1.30; 1H-20); (0.98 *s*, 6.1; 3H-21); (1.70; 1.40; 2H-22); (3.61 *m*; 1H-23); (2.68 *d*,8.3; 1H-24); (1.35 *s*; 3H-26); (1.34 *s*; 3H-27); (1.07 *s*; 3H-28); (1.14 *s*; 3H-29); (1.04 *s*; 3H-30).

*Dihydroniloctin* (**3**): ^13^C *δ* (ppm): 37.19 (CH_2_-1); 27.66 (CH_2_-2); 79.25 (CH-3); 38.96 (C-4); 50.61 (CH-5); 23.94 (CH_2_-6); 118.05 (CH-7); 145.57 (C-8); 48.91 (CH-9); 34.93 (C-10); 18.09 (CH_2_-11); 33.97 (CH_2_-12); 43.58 (C-13); 51.79 (C-14); 33.78 (CH_2_-15); 28.79 (CH_2_-16); 53.25 (CH-17); 13.11 (CH_3_-18); 21.71 (CH_3_-19); 33.59 (CH-20); 19.84 (CH_3_-21); 40.69 (CH_2_-22); 69.30 (CH-23); 68.51 (CH-24); 60.34 (C-25); 19.13 (CH_3_-26); 24.88 (CH_3_-27); 27.62 (CH_3_-28); 14.71 (CH_3_-29); 27.24 (CH_3_-30). ^1^H *δ* (ppm): (3.24 *dd* 11.1, 4.1; 1H-3); (5.26 *sl*; 1H-7); (0.75 *s*; 3H-18); (0.82 *s*; 3H-19); (0.96 *d*, 6.4; 3H-21); (3.57 *m*; 1H-23); (2.66 *d*, 8.2; 1H-24); (1.32 *s*; 3H-26); (1.33 *s*; 3H-27); (0.96 *s*, 3H-28); (0.86 *s*; 3H-29); (0.97 *s*; 3H-30).

*Melianone* (**4**): ^13^C *δ* (ppm): 38.49 (CH_2_-1); 34.91 (CH_2_-2); 216.96 (C-3); 47.88 (C-4); 52.35 (CH-5); 24.38 (CH_2_-6); 118.35 (CH-7); 145.58 (C-8); 48.37 (CH-9); 35.10 (C-10); 17.72 (CH_2_-11); 31.60 (CH_2_-12); 43.76 (C-13); 51.03 (C-14); 33.93 (CH_2_-15); 27.64 (CH_2_-16); 49.37 (CH-17); 22.62 (CH_3_-18); 12.75 (CH_3_-19); 47.04 (CH-20); 101.19 (CH-21) e 97.74 (CH-21); 34.83 (CH_2_-22); 77.50 (CH-23); 65.37 (CH-24); 57.30 (C-25); 24.92 (CH_3_-26); 19.43 (CH_3_-27); 24.44 (CH_3_-28); 21.59 (CH_3_-29); 27.28 (CH_3_-30). ^1^H *δ* (ppm): (1.98; 1.45; 2H-1); (2.77 *dt* 14.5, 5.4; H-2); (2.25 *dt* 14.5, 4.0; H-2) ; (1.73; H-5); (2.15; 2H-6); (5.34; H-7); ( 2.30 H-9); (1.62; 2H-11); (2.05; 1.75; 2H-12); (1.55; 2H-15); (1.95; 1.86; 2H-16); (1.82; H-17); (0.91 *s*; 3H-18); (1.02 *s*; 3H-19); (2.25; H-20); (5.38 *dl* 3.0; H-21); (2.15; 1.40; 2H-22); (3.95 *m*; H-23); (2.72 *d* 7.6; H-24); (1.34 *s*, 3H-26); (1.32 *s*; 3H-27); (1.06 *s*; 3H-28); (1.13 *s*; 3H-29); (1.05 *s*; 3H-30).

*Piscidinol* A (**6**): ^13^C *δ* (ppm): 38.25 (CH_2_-1); 34.98 (CH_2_-2); 47 (C-4); 52.31 (CH-5); 24.37 (CH_2_-6); 117.95 (CH -7); 145 (C-8); 48.46 (CH-9); 35 (C-10); 18.31 (CH_2_-11); 33.99 (C-12); 43 (C-13); 51 (C-14); 33.77 (CH_2_-15); 28.48 (CH_2_-16); 53.60 (CH -17); 22.06 (CH_3_-18); 12.80 (CH_3_-19); 33.69 (CH-20); 18.92 (CH_3_-21); 40.40 (CH_2_-22); 69.71 (CH-23); 74.81 (CH-24); 72 (C-25); 26.23 (CH_3_-26); 27.50 (CH_3_-27); 24.39 (CH_3_-28); 21.61 (CH_3_-29); 27.41 (CH_3_-30). ^1^H *δ* (ppm): (2.05; 1.52; 2H-1); (2.78 *dt* 14.7, 5.7; H-2); (2.27 *dt* 14.7, 3.8; H-2); (1.75 *t 8.9*; H-5); (2.15 *m*; 2H-6); (5.34 *tl* 3.1; H-7); (2.40 *m*; H-9); (1.60; 2H-11); (1.85; 1.52; 2H-12); (1.70; 1.45; 2H-15); (1.90; 1.25; 2H-16); (1.71; H-17); (0.85 *s*; 3H-18); (1.03 *s*; 3H-19); (1.48; H-20); (0.96 *d* 6.4; 3H-21); (1.90; 1.25; 2H-22); (4.15 *dd* 9.7, 5.0; H-23); (3.10 *sl*; H-24); (1.36 *s*; 3H-26); (1.39 *s*; 3H-27); (1.07 *s*; 3H-28); (1.19 s; 3H-29); (1.04 *s*; 3H-30).

*Melianone lactone* (**7**): ^13^C *δ* (ppm): 38.51 (CH_2_-1); 34.90 (CH_2_-2); 216.76 (C-3); 47.88 (C-4); 52.57 (CH-5); 24.39 (CH_2_-6); 118.33 (CH-7); 145.87 (C-8); 47.37 (CH-9); 35.12 (C-10); 17.64 (CH_2_-11); 31.02 (CH_2_-12); 43.91 (C-13); 50.53 (C-14); 33.89 (CH_2_-15); 24.22 (CH_2_-16); 48.49 (CH-17); 23.44 (CH_3_-18); 12.73 (CH_3_-19); 40.85 (CH-20); 178.04 (C-21); 30.21 (CH_2_-22); 77.96 (CH-23); 64.48 (CH-24); 57.54 (C-25); 24.82 (CH_3_-26); 19.47 (CH_3_-27); 24.52 (CH_3_-28); 21.55 (CH_3_-29); 27.59 (CH_3_-30) ^1^H *δ* (ppm): (2.10; 1.48; 2H-1); (2.60; 2.30; 2H-2); (1.51; H-3); (2.15; 1.95; 2H-6); (5.35 *sl*; H-7); (2.40; H-9); (1.60; 2H-11); (1.85; 1.75; 2H-12); (1.60; 2H-15); (1.90; 1.45; 2H-16); (2.17; H-17); (0.86 *s*; 2H-18); (1.05 *s*; 2H-19); (2.72; H-20); (2.35; 1.85; 2H-22); (4.18; H-23); (2.84; H-24); (1.40 *s*; 2H-26); (1.37 *s*; 3H-27); 1.07 *s*; 3H-28); (1.14 *s*; 3H-29); (1.09 *s*; 3H-30).

*Hirtin* (**8**): ^13^C *δ* (ppm): 150.52 (CH-1); 125.84 (CH-2); 195.66 (C-3); 60.43 (C-4); 129.1 (C-5); 141.90 (C-6); 196.19 (C-7); 46.15 (C-8); 42.01 (CH-9); 39.85 (C-10); 72.41 (CH-11); 77.76 (CH-12); 45.32 (C-13); 67.59 (C-14); 55.15 (CH-15); 32.15 (CH_2_-16); 41.37 (CH-17); 15.80 (CH_3_-18); 25.97 (CH_3_-19); 121.48 (C-20); 140.48 (CH-21); 111.22 (CH-22); 142.69 (CH-23); 22.85 (CH_3_-28); 170.06 (C-29); 22.52 (CH_3_-30); 174.49 (C-1′); 34.10 (CH-2′); 18.70 (MeO-3′); 18.68 (MeO-4′); 169.39 (C-1′′); 21.20 (MeO-2′′). ^1^H *δ* (ppm): (7.0 *d* 10.1; H-1); (6.19 *d* 10.1; H-2); (2.99 *s*; H-9); (5.37 *s*; H-11); (5.23 *s*; H-12); (2.93 *s*; H-15); (2.33 *dd* 13.8, 6.7; 2H-16); (2.02 *dd* 13.8, 11.0; 2H-16); (2.96 *dd* 11.0, 6.7; H-17); (0.84 *s*; 3H-18); (1.45 *s*; 3H-19); (7.15 *sl*; H-21); (6.2 *sl*; H-22); (7.32 *t* 1.6; H-23); (1.85 *s*; 3H-28); (2.47 *sept* 7.0; H-2′); (2.31 *s*; H2′′-MeO); (1.10 *d* 7.0; H3′-MeO); 0.98 *d* 7.0; H4′-MeO); (1.43 *s*; 3H-30); (6.47 *s*; H-HO).

*Spathulenol* (**9**): ^13^C *δ* (ppm): 53.42 (CH-1); 26,73 (CH_2_-2); 41.74 (CH_2_-3); 80 (CH-4); 54.33 (CH-5); 29.91 (CH-6); 27.48 (CH-7); 24.77 (CH_2_-8); 38.86 (CH_2_-9); 153,40 (C-10); 20 (C-11); 16.15 (CH_3_-12); 28.69 (CH_3_-13); 108.28 (CH_2_-14); 26.10 (CH_3_-15). ^1^H *δ* (ppm): (2.22 *m*; H-1); (2.08 *m*; 1.90 *m*; 2H-2); (1.80 *m*; 1.60 *m*;2H-3); (1.30 *t*3.5; H-5); (0.49 *dd*, *J* = 11.3, 9.5 Hz; H-6); (0.74 *m*; H-7); (1.70 *m*; 2H-8); (2.4 *dd*, *J* = 12.3, 5.3 Hz, 2H-9); (1.06 *s*, 3H-12); (1.08 s, 3H-13); (4.71 *s*; 4.69 *s*; 2H-14); (1.32 *s*; 3H-15).

## 4. Conclusions

The hexane extract from the fruits of *T. hirta* provided five protolimonoids **2**–**7**, one tertranortriterpenoid **8** and one sesquiterpene **9**, which were isolated in a previous phytochemical investigation. A cycloartane-type triterpene **1**, named hirtinone, is described for the first time in the literature.

## Supplementary Materials

Supplementary materials can be accessed at: http://www.mdpi.com/1420-3049/18/3/2589/s1.
