# Supplementary material for: Hirtinone, a Novel Cycloartane-Type Triterpene and Other Compounds from Trichilia hirta L. (Meliaceae)
Source: Molecules. 2013 Feb 26;18(3):2589–97. doi: 10.3390/molecules18032589 (PMC6270332; doi:10.3390/molecules18032589)

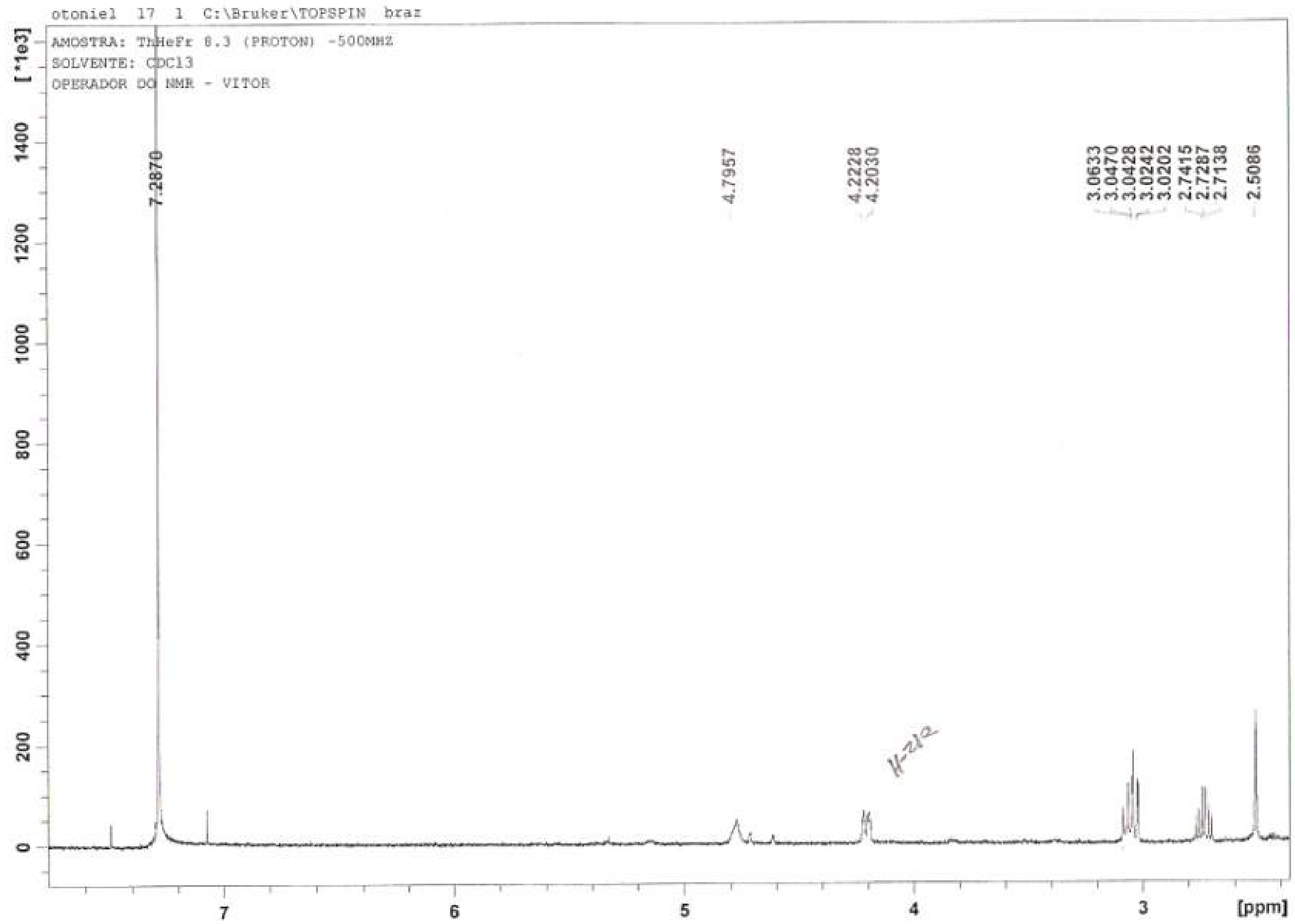

otoniel 17 1 C:\Bruker\TOPSPIN braz
AMOSTRA: ThHeFr 8.3 (PROTON) -500MHZ
SOLVENTE: CDCl3
OPERADOR DO NMR - VITOR
[*1e3]
1400
1200
1000
800
600
400
200
0
7.2870
4.7957
4.2228
4.2030
3.0633
3.0470
3.0428
3.0242
3.0202
2.7415
2.7287
2.7138
2.5086
7
6
5
4
3
[ppm]

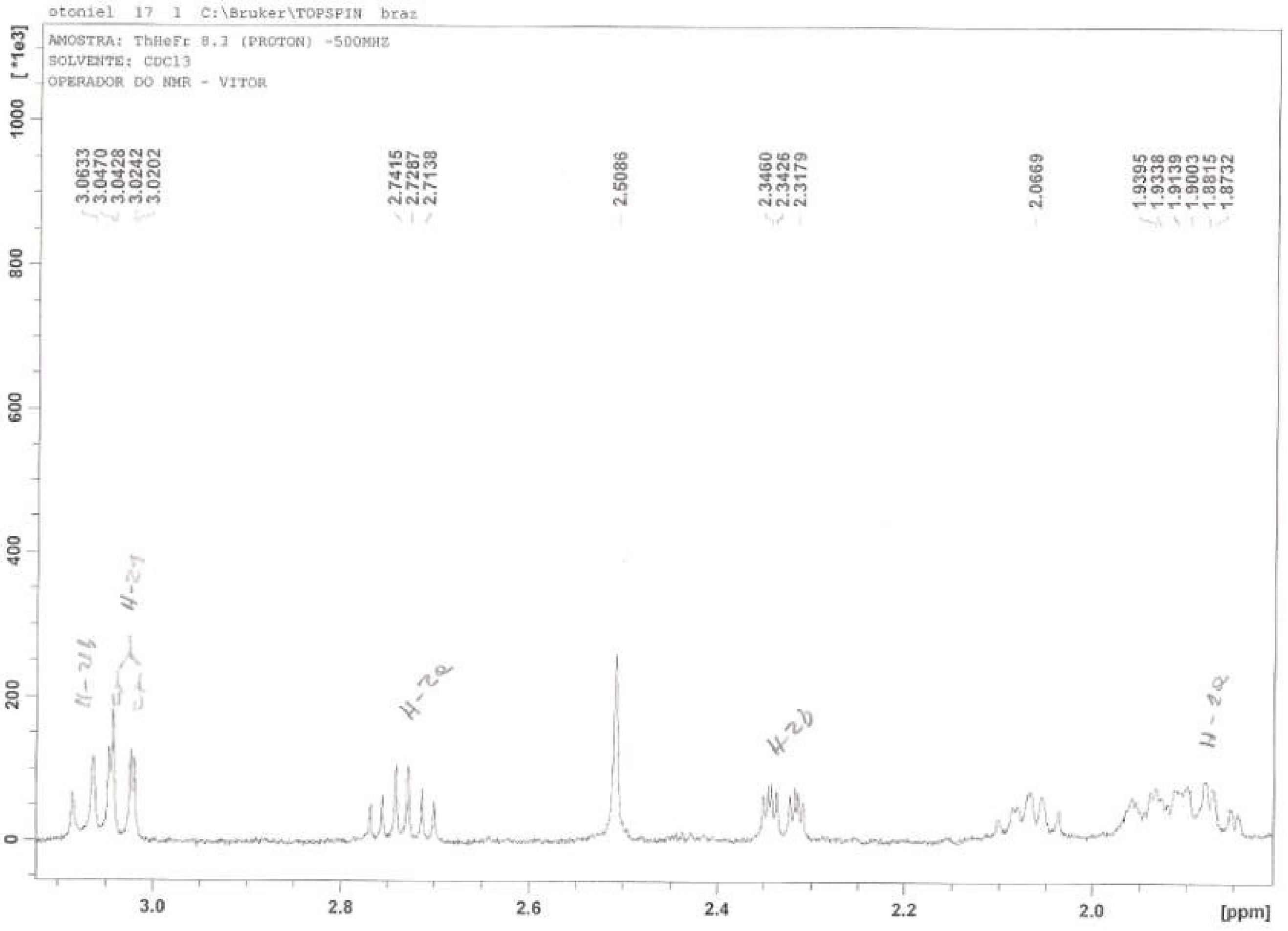

otoniel 17 1 C:\Bruker\TOPSPIN braz
AMOSTRA: ThHeFr 8.3 (PROTON) -500MHZ
SOLVENTE: CDCl3
OPERADOR DO NMR - VITOR
[*1e3]
1000
800
600
400
200
0
3.0633
3.0470
3.0428
3.0242
3.0202
2.7415
2.7287
2.7138
2.5086
2.3460
2.3426
2.3179
2.0669
1.9395
1.9338
1.9139
1.9003
1.8815
1.8732
H-21
H-2e
H-2b
3.0
2.8
2.6
2.4
2.2
2.0
[ppm]

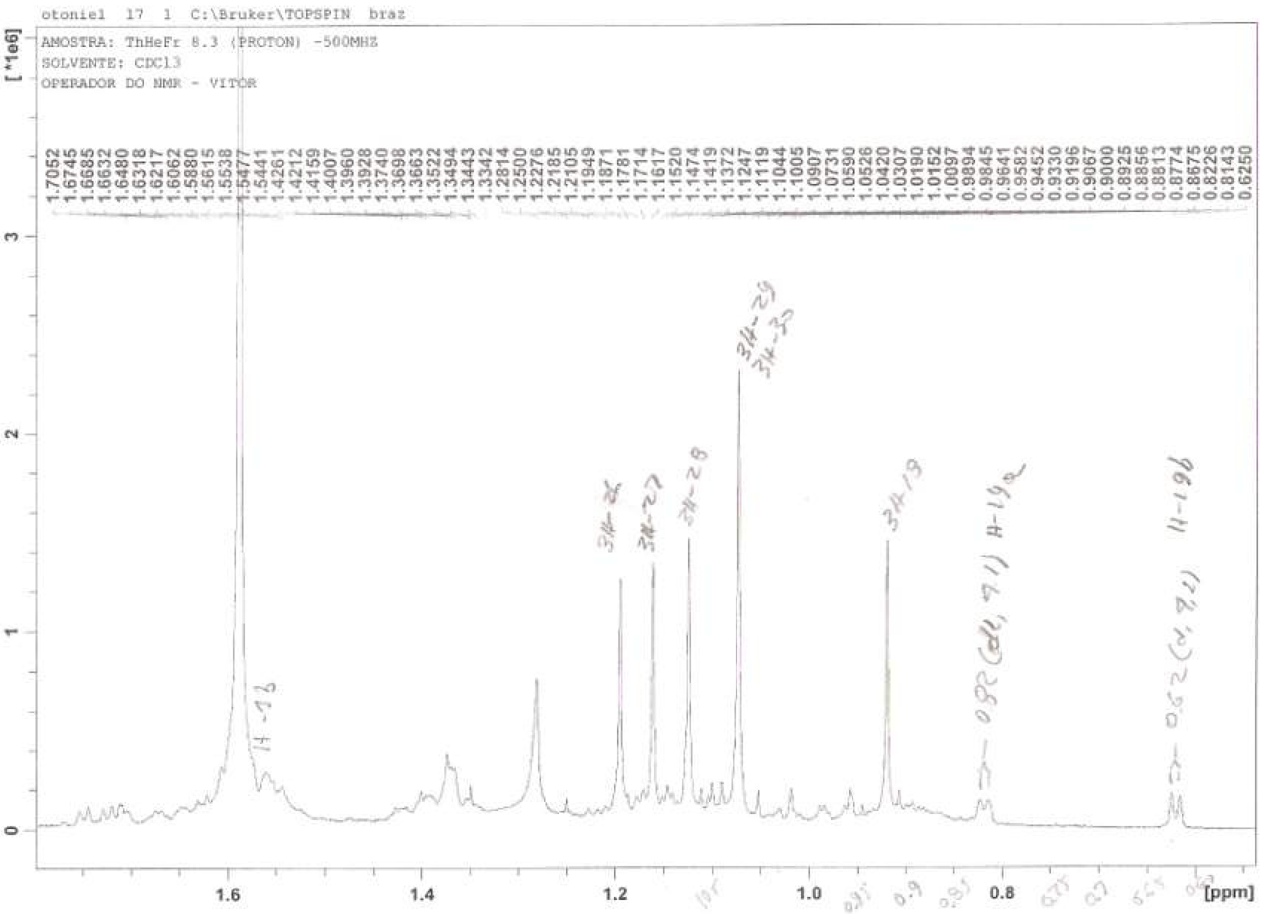
otoniel 17 1 C:\Bruker\TOPSPIN braz
AMOSTRA: ThHeFr 8.3 (PROTON) -500MHZ
SOLVENTE: CDCl3
OPERADOR DO NMR - VITOR
[*1e6]
1.7052 1.6745 1.6685 1.6632 1.6480 1.6318 1.6217 1.6062 1.5880 1.5615 1.5538 1.5477 1.5441 1.4261 1.4212 1.4159 1.4007 1.3960 1.3928 1.3740 1.3698 1.3663 1.3522 1.3494 1.3443 1.3342 1.2814 1.2500 1.2276 1.2185 1.2105 1.1949 1.1871 1.1781 1.1714 1.1617 1.1520 1.1474 1.1419 1.1372 1.1247 1.1119 1.1044 1.1005 1.0907 1.0731 1.0590 1.0526 1.0420 1.0307 1.0190 1.0152 1.0097 0.9894 0.9845 0.9641 0.9582 0.9452 0.9330 0.9196 0.9067 0.9000 0.8925 0.8856 0.8813 0.8774 0.8675 0.8226 0.8143 0.6250
H-1b
3H-26
3H-27
3H-28
3H-29
3H-30
3H-19
0.82 (dd, 7.1) H-19a
0.62 (d, 7.2) H-19b
1.6
1.4
1.2
1.0
0.8
[ppm]

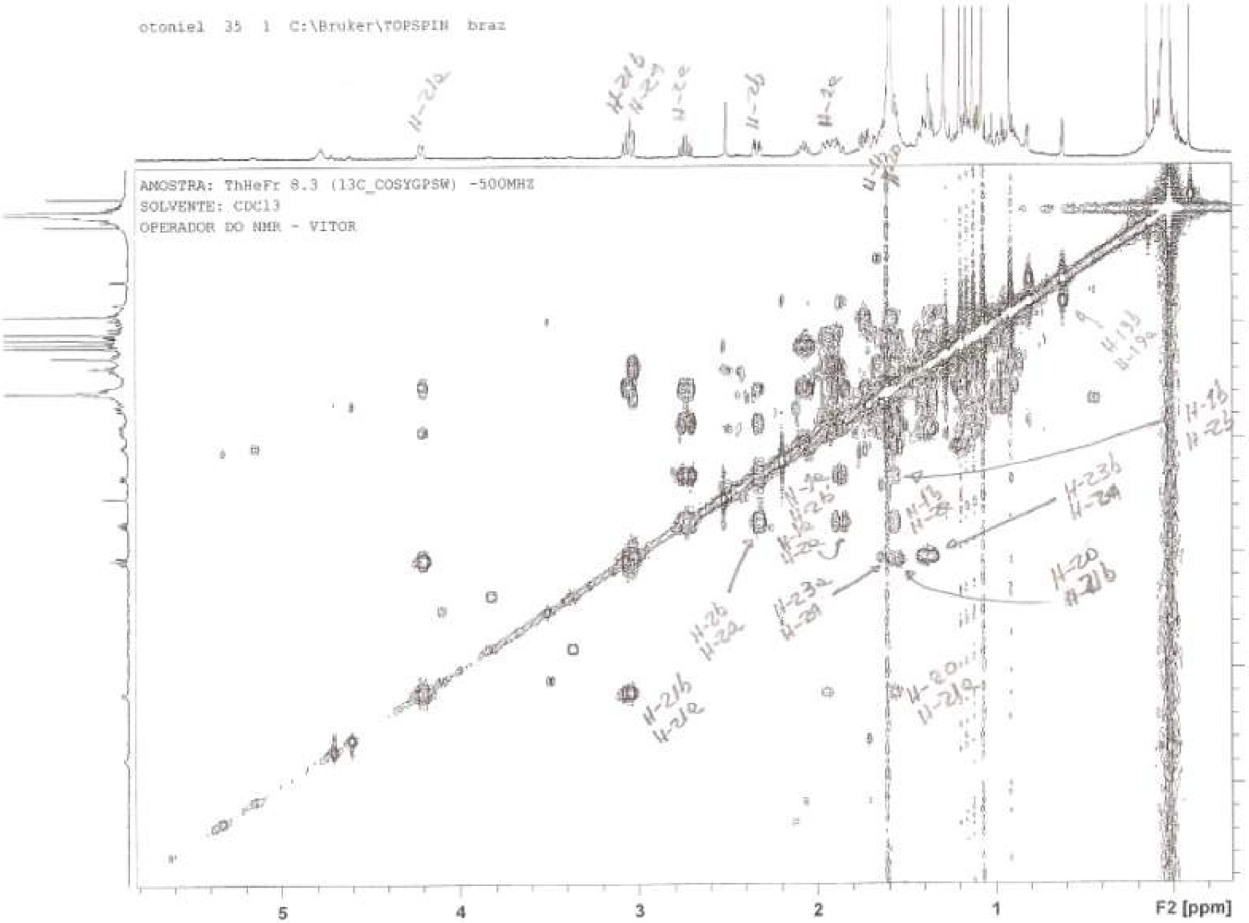
otoniel 35 1 C:\Bruker\TOPSPIN braz
AMOSTRA: ThHeFr 8.3 (13C_COSYGPSW) -500MHZ
SOLVENTE: CDCl3
OPERADOR DO NMR - VITOR
F1 [ppm]
F2 [ppm]

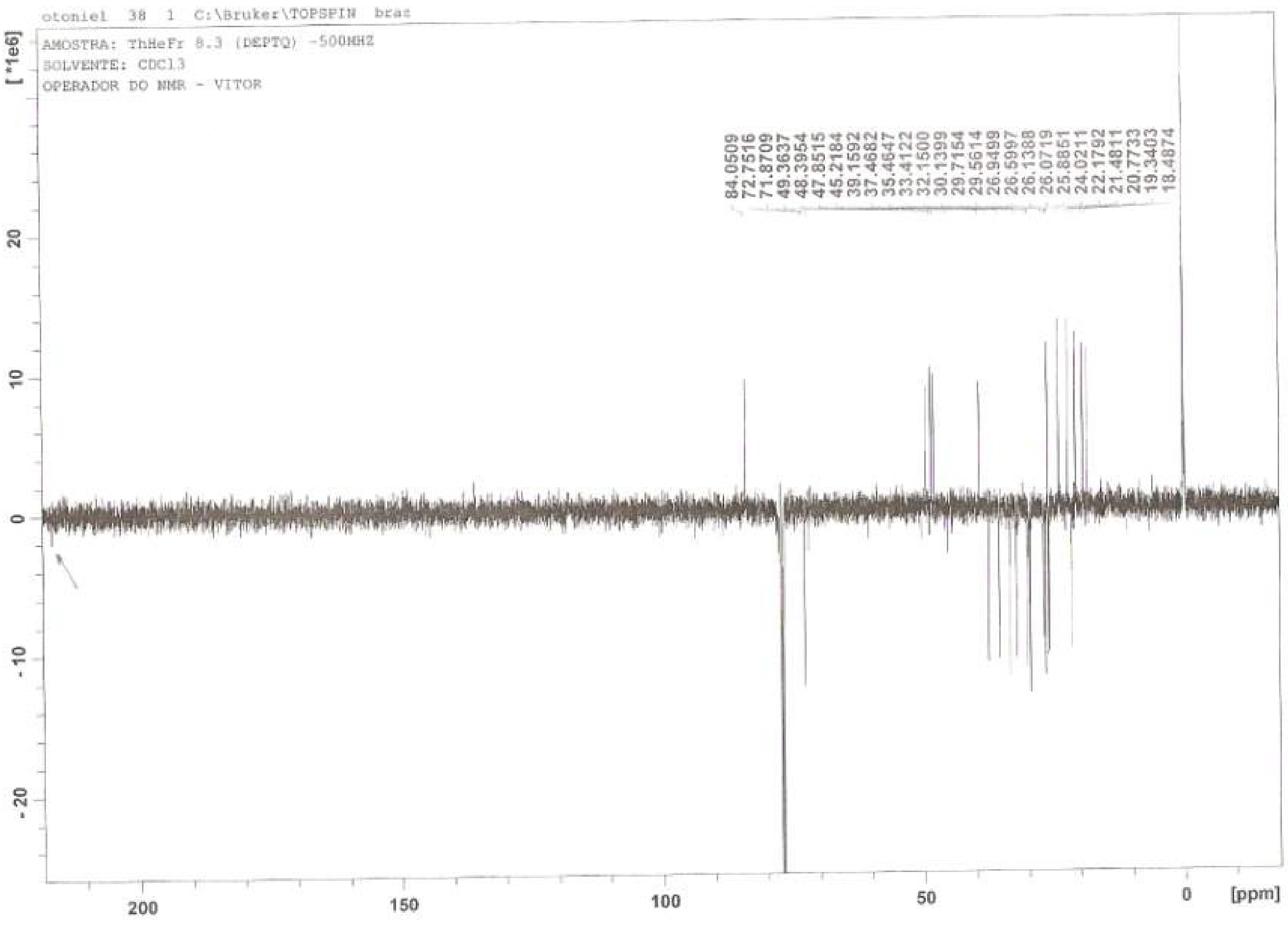

otoniel 38 1 C:\Bruker\TOPSPIN bras
AMOSTRA: ThHeFr 8.3 (DEPTQ) -500MHZ
SOLVENTE: CDCl3
OPERADOR DO NMR - VITOR
[*1e6]
20
10
0
- 10
- 20
84.0509
72.7516
71.8709
49.3637
48.3954
47.8515
45.2184
39.1592
37.4682
35.4647
33.4122
32.1500
30.1399
29.7154
29.5614
26.9499
26.5997
26.1388
26.0719
25.8851
24.0211
22.1792
21.4811
20.7733
19.3403
18.4874
200
150
100
50
0
[ppm]

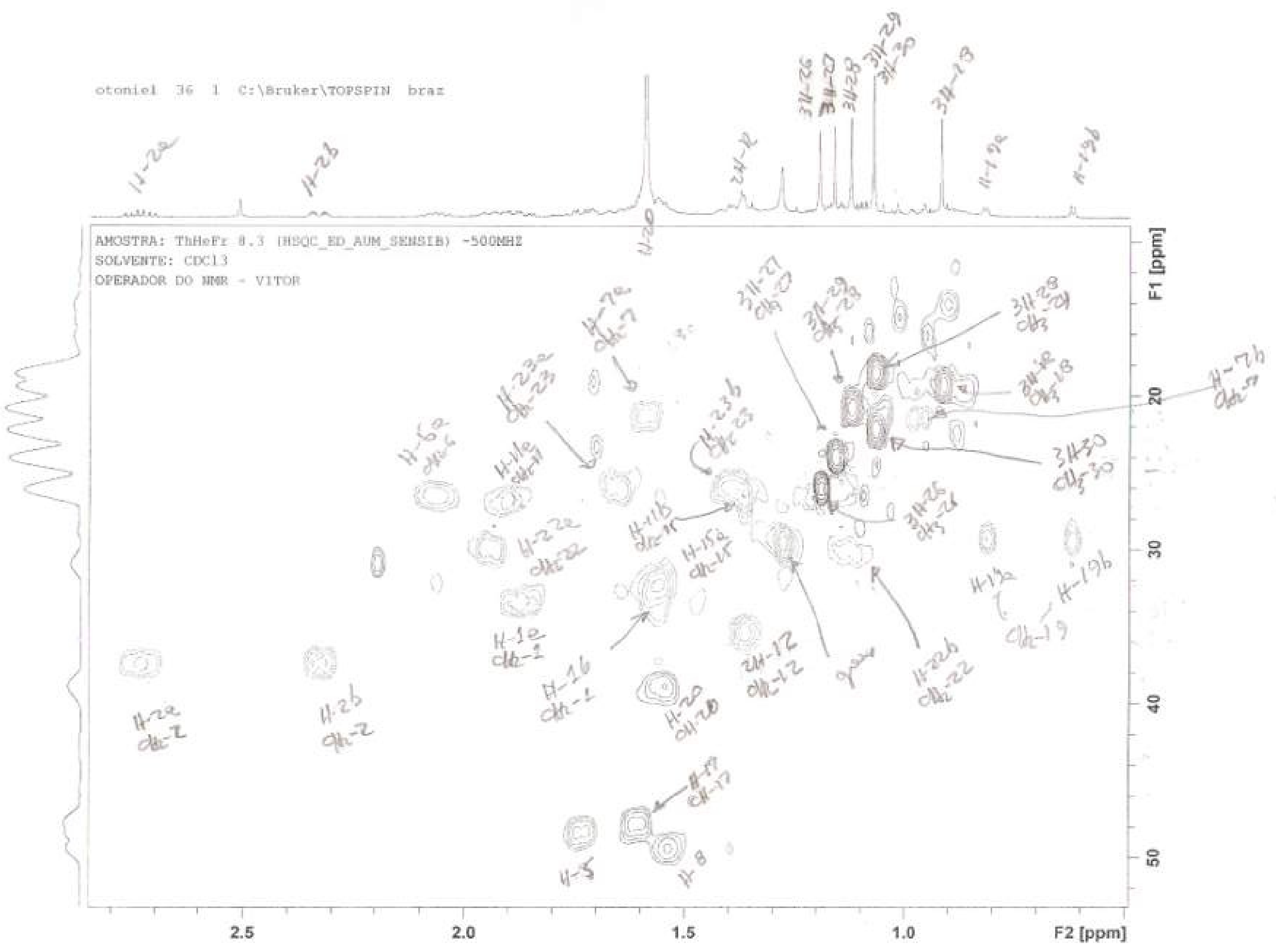

otoniel 36 1 C:\Bruker\TOPSPIN braz
AMOSTRA: ThHeFr 8.3 (HSQC_ED_AUM_SENSIB) -500MHZ
SOLVENTE: CDCl3
OPERADOR DO NMR - VITOR
2.5
2.0
1.5
1.0
F2 [ppm]
20
30
40
50
F1 [ppm]

otoniel 47 1 C:\Bruker\TOPSPIN braz

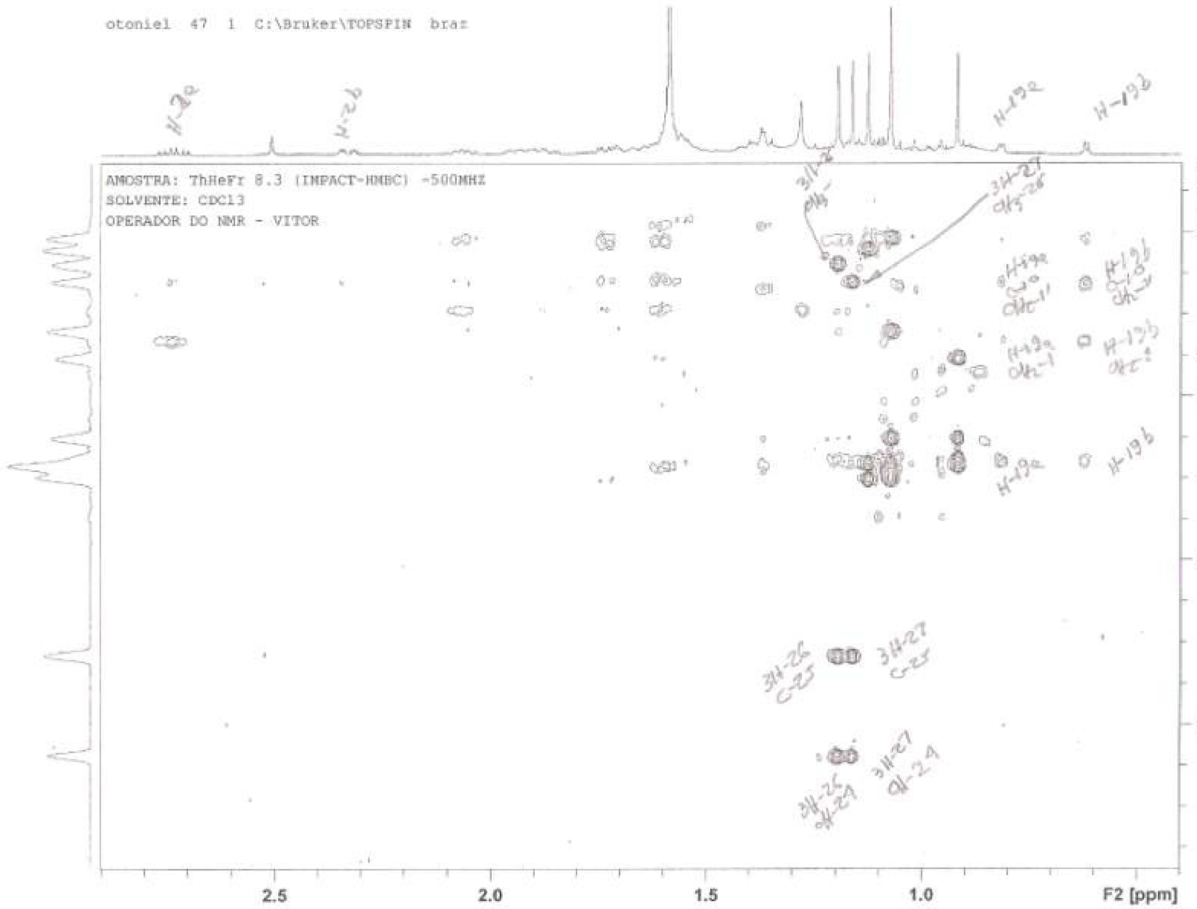

Supplement: Supplementary file 1 [file molecules-18-02589-s001.pdf]
